# The Successful Management of a Life-Threatening Anterior Mediastinal Tumor Using Extracorporeal Membrane Oxygenation and Multidisciplinary Intervention: A Case Report

**DOI:** 10.7759/cureus.88637

**Published:** 2025-07-23

**Authors:** Junya Fuchigami, Shingo Hamaguchi, Mumon Takita, Hiroshi Handa, Fumiaki Sano

**Affiliations:** 1 Department of Diagnostic and Interventional Radiology, St. Marianna University School of Medicine, Kawasaki, JPN; 2 Department of Emergency and Critical Care Medicine, St. Marianna University School of Medicine, Kawasaki, JPN; 3 Department of Respiratory Diseases, St. Marianna University School of Medicine, Kawasaki, JPN; 4 Department of Hematology and Oncology, St. Marianna University School of Medicine, Kawasaki, JPN

**Keywords:** airway obstruction, airway stent, anterior mediastinal tumor, anticoagulation, core needle biopsy, extracorporeal membrane oxygenation, mediastinal large b-cell lymphoma, multidisciplinary intervention

## Abstract

Primary mediastinal large B-cell lymphoma (PMBCL) is a rare and potentially life-threatening tumor, especially when it causes compression of vital thoracic structures. Rapid diagnosis and timely treatment are essential, but diagnostic procedures may be complicated by severe airway obstruction. We report a case of a previously healthy 23-year-old female who presented with progressive orthopnea and facial edema over several weeks. Imaging revealed a massive anterior mediastinal tumor compressing the carina, main bronchi, and pulmonary arteries. A multidisciplinary team was involved early in the evaluation and discussed potential management strategies. Although initially stable, the patient developed shock on the second hospital day. Venoarterial extracorporeal membrane oxygenation (ECMO) was initiated to provide cardiopulmonary support and facilitate safe diagnostic intervention. Once hemodynamic and respiratory stability were achieved, endotracheal intubation and ultrasound-guided core needle biopsy (CNB) were performed. Anticoagulation was withheld until post-biopsy bleeding risk was ruled out. Rapid on-site evaluation suggested malignant lymphoma, and chemotherapy was initiated promptly. The tumor regressed rapidly, allowing for ECMO weaning on day nine. The patient was discharged without complications, and the final diagnosis established was PMBCL.

We successfully provided prompt treatment for a large anterior mediastinal tumor causing respiratory and circulatory failure through a multidisciplinary approach, including ECMO support and rapid CNB diagnosis. Although this strategy may not be appropriate for tumors unresponsive to chemotherapy, it may be effective in cases of tumors likely to respond to chemotherapy, such as lymphoma. This report highlights the practical steps and clinical strategies that may guide the management of similar cases in emergency or critical care settings.

## Introduction

Mediastinal tumors can compress the trachea, lungs, and heart, and may become life-threatening [1]. Primary mediastinal large B-cell lymphoma (PMBCL) was previously classified as a subtype of diffuse large B-cell lymphoma (DLBCL) but is now recognized as a distinct entity based on gene expression profiling. PMBCL accounts for approximately 7% of DLBCLs and 2-4% of all non-Hodgkin lymphomas [2]. It mainly affects young women and typically presents as a rapidly enlarging anterior mediastinal mass. PMBCL generally responds well to chemotherapy, with a five-year survival rate of approximately 85% [3]. Extracorporeal membrane oxygenation (ECMO) is an advanced life support system that temporarily replaces cardiac and/or pulmonary function through an external circuit. ECMO is associated with risks such as bleeding, thrombosis, infection, and hemolysis. Therefore, patients must be selected carefully [4]. In patients with anterior mediastinal tumors causing severe airway compression, the diagnostic process itself can be hazardous due to the risk of respiratory or circulatory failure. In such situations, ECMO may help maintain physiological stability and allow for a safe transition to treatment. We report a case of a patient in whom we successfully performed percutaneous needle biopsy and chemotherapy under ECMO support, thereby saving the patient's life from impending airway obstruction caused by a massive PMBCL.

## Case presentation

**Figure 1:**
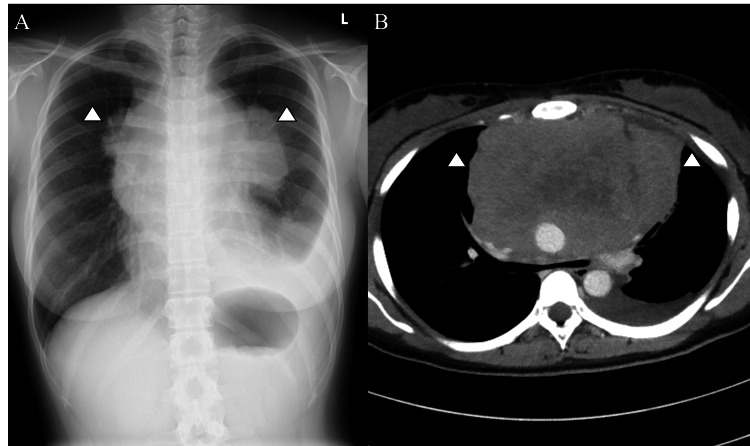
Imaging findings on the day of admission A. Chest radiograph on the day of admission. B. Contrast-enhanced CT on the day of admission The images showed a massive anterior mediastinal tumor with internal necrosis compressing the carina, bilateral main bronchi, and pulmonary arteries CT: computed tomography

A previously healthy 23-year-old female presented to a general hospital with progressive orthopnea and facial edema for several weeks. Chest radiography and contrast-enhanced CT revealed a large anterior mediastinal mass, as shown in Figures 1A, 1B. A massive anterior mediastinal tumor compressed the carina, bilateral main bronchi, and pulmonary arteries. The patient was transferred to our hospital in the evening for airway management, evaluation, and treatment. At presentation, the patient was ambulatory and exhibited no respiratory distress while sitting and maintaining oxygenation. However, the airway compression symptoms worsened when the patient was placed in the supine position. Emergency blood tests revealed a white blood cell count of 6,100/μL, hemoglobin of 11.8 g/dL, platelet count of 390,000/μL, and lactate dehydrogenase (LDH) of 865 U/L. The other biochemical and coagulation parameters were within normal limits. The respiratory medicine team initially considered airway stenting and bronchoscopic biopsy. However, after multidisciplinary consultation with thoracic surgeons and anesthesiologists, based on CT findings, the procedures were regarded as high risk due to the likelihood of complete airway obstruction under general anesthesia. In addition, there was a concern that the tumor was so massive that even if an airway stent was inserted, sufficient expansion might not be achieved. As her condition was stable, she was admitted to the high-care unit for overnight monitoring. Further, multidisciplinary discussions were planned for the following morning to determine the treatment strategy.

On the morning of day two of hospitalization, the patient went into shock, with a blood pressure of 58/36 mmHg, and was given fluid resuscitation along with continuous norepinephrine infusion, achieving a mean arterial pressure above 65 mmHg. Further multidisciplinary discussions involving oncology, hematology, emergency medicine, respiratory medicine, thoracic surgery, radiology, and anesthesiology were conducted. Based on the patient’s age and clinical course, malignant lymphoma or germ cell tumors were suspected. Laboratory evaluation performed in the morning revealed an elevated interleukin-2 level of 2,909 U/mL, while human chorionic gonadotropin and alpha-fetoprotein levels were within normal limits, raising suspicion of malignant lymphoma. With the expectation of tumor shrinkage following chemotherapy, a percutaneous needle biopsy was planned under venoarterial ECMO support.

**Figure 2:**
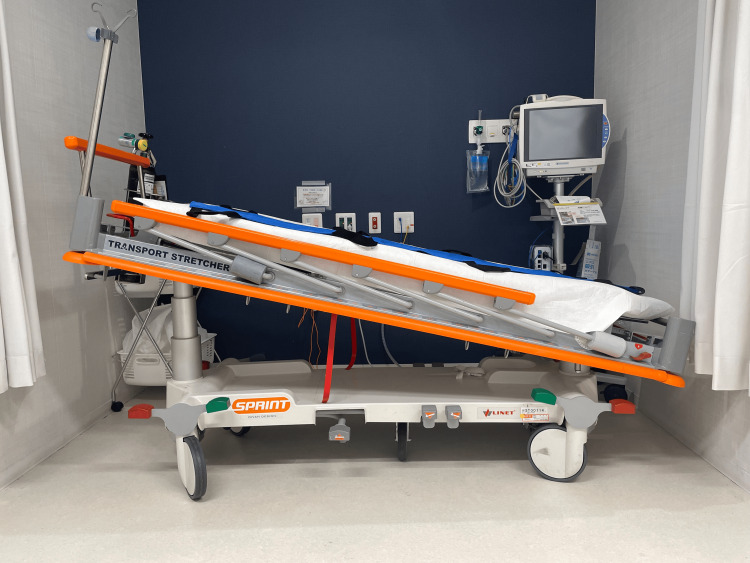
Adjustable transport stretcher An adjustable transport stretcher was used to elevate the head while maintaining hip joint extension and facilitating femoral puncture

With the patient awake and positioned at a 30° head-up tilt on an adjustable transport stretcher (SPRINT; LINET Group SE, Dordrecht, Netherlands), as illustrated in Figure 2, while avoiding flexion at the hip joints as much as possible, emergency physicians inserted a 5-Fr × 10-cm sheath into the right femoral vein and a 4-Fr × 10-cm sheath into the left femoral artery under ultrasound guidance. The patient was then transferred to a hybrid emergency room system (HERS). Maintaining the same head-up position, the sheaths were exchanged over the guidewires under fluoroscopic guidance for the ECMO cannulas. Venoarterial ECMO was established with drainage through the right femoral vein, with the cannula tip placed in the suprahepatic inferior vena cava, and reinfusion through the left femoral artery, with the cannula tip positioned in the left common iliac artery. Left distal limb perfusion was maintained by inserting a 4-Fr × 10-cm sheath antegradely into the left femoral artery and connecting it to a side port of the arterial ECMO system. Once ECMO stabilized respiratory and circulatory functions, sedation and endotracheal intubation were performed. Subsequently, percutaneous needle biopsy was performed by interventional radiologists under ultrasound guidance. After confirming the absence of active bleeding following biopsy, low-dose systemic anticoagulation was initiated with a target activated partial thromboplastin time (APTT) of approximately 45 s. Rapid on-site evaluation (ROSE) suggested malignant lymphoma, and R-CHOP (rituximab, cyclophosphamide, doxorubicin, vincristine, prednisone) chemotherapy was initiated the following day.

**Figure 3:**
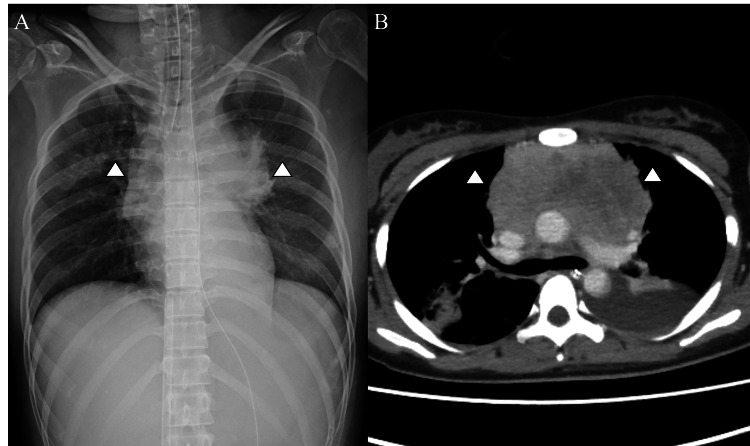
Imaging results on hospital day 9 A. Portable chest radiograph on hospital day 9. B. Contrast-enhanced CT on hospital day 9 The anterior mediastinal mass was reduced in size, resulting in the resolution of compression on the trachea, main bronchi, and pulmonary arteries. Consolidation was noted in the right upper lobe, likely representing airway-related inflammation such as pneumonia CT: computed tomography

On day nine of hospitalization, chest radiography and contrast-enhanced CT (Figures 3A, 3B) confirmed significant tumor reduction and decreased compression of the airway and pulmonary arteries. ECMO was successfully discontinued on the same day. The patient was discharged on day 37 without complications.

**Figure 4:**
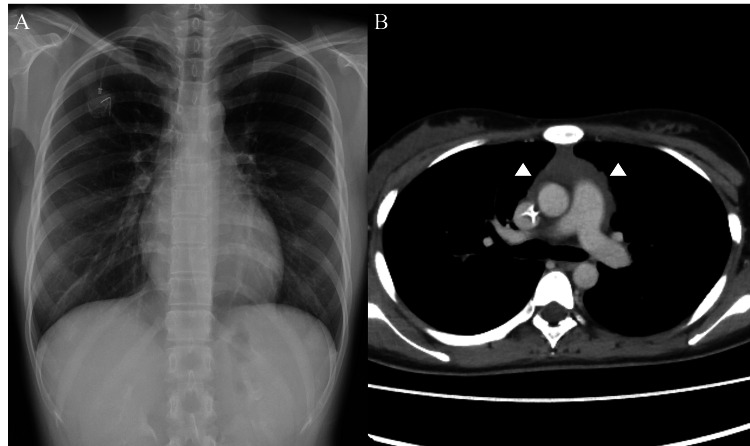
Imaging findings after the completion of four cycles of DA-EPOCH-R A. Chest radiograph after the completion of four cycles of DA-EPOCH-R. B. Contrast-enhanced CT after the completion of four cycles of DA-EPOCH-R The anterior mediastinal mass regressed significantly and was scarcely recognizable on chest radiography CT: computed tomography: DA-EPOCH-R: dose-adjusted etoposide, prednisone, vincristine, cyclophosphamide, doxorubicin, and rituximab

The definitive pathological diagnosis was later established as PMBCL. Chest radiography and CT imaging after four cycles of DA-EPOCH-R (dose-adjusted etoposide, prednisone, vincristine, cyclophosphamide, doxorubicin, and rituximab) chemotherapy (following one cycle of R-CHOP) showed significant tumor shrinkage (Figures 4A, 4B). The patient remains well and continues to receive treatment.

## Discussion

In women aged 10-39 years, the most common anterior mediastinal tumor is lymphoma, specifically, Hodgkin’s disease or mediastinal large-cell non-Hodgkin lymphoma. Following lymphoma, thymomas and benign teratomas are the most common anterior mediastinal tumors in this age group. If these diagnoses are not suspected, assessing alpha-fetoprotein and beta-human chorionic gonadotropin levels may help evaluate the rare but possible presence of non-seminomatous germ cell tumors [5].

Airway stents are usually classified as either metallic or silicone. Metal stents are simpler to place and adapt well to airway anatomy, but are harder to remove. Silicone stents are easier to take out and can be customized during the procedure; however, they require rigid bronchoscopy and are more likely to migrate [6]. Hybrid stents designed to incorporate the advantages of both types have also been developed [7]. In our cases, where tumor shrinkage is expected after additional treatment, either silicone or hybrid stents can be used, considering that stent removal may be required later. Silicone stent placement requires general anesthesia. Our patient presented with symptoms of severe airway obstruction, including orthopnea and facial edema, and CT revealed significant airway compression.

Complications during induction or intubation are most often seen in young adults with rapidly growing tumors, such as lymphoma, when symptoms of severe airway obstruction are present [8]. In children, mediastinal masses causing more than one-third tracheobronchial compression have been reported to carry a high risk of complete airway obstruction under general anesthesia [9]. In Japan, only straight-type hybrid stents are available. Therefore, multiple stents must be placed to cover the lesion, which increases the risk of stent migration. Considering the risks of airway obstruction, insufficient stent expansion due to the massive tumor, and potential stent migration, a multidisciplinary discussion led to the decision to initiate temporary venovenous ECMO support based on the suspicion of lymphoma and the presumed chemosensitivity of the tumor. Venoarterial ECMO was initiated as the patient developed circulatory failure. Several cases of hematological malignancies in the anterior mediastinum requiring ECMO support have been reported [10,11].

We highlight the three key strategies implemented in this case. First, large anterior mediastinal tumors with airway compression often prevent patients from tolerating the supine position, thereby increasing the technical difficulty of invasive procedures. Although biopsy in a seated position might have been technically possible, the posture would have been unstable, making it challenging to manage potential complications, such as transcatheter arterial embolization. Therefore, ECMO was initiated in advance not only to support circulatory and respiratory failure but also to ensure a stable environment for biopsy and allow for prompt intervention in case of complications. In this case, a stretcher with adjustable angles was used to maintain the safest upright posture. Positioning the patient in the reverse Trendelenburg position minimized hip joint flexion, thereby facilitating optimal vascular access.

Second, to minimize bleeding risk during biopsy, ECMO was initiated without systemic anticoagulation until active bleeding related to biopsy complications was ruled out. ECMO without anticoagulation is reportedly safe for airway surgery and trauma [12,13]. In a single-center retrospective analysis, Wood et al. demonstrated that VA-ECMO can be managed without systemic anticoagulation for a median duration of 70 h. Although the incidences of thrombotic and hemorrhagic complications tended to be lower in the anticoagulation-free group compared to the anticoagulation group, the differences were not significant [14].

Third, a smaller-gauge biopsy needle was preferred to reduce the risk of bleeding. However, a larger amount of tissue is required for the diagnosis of lymphoma to evaluate the whole histological architecture, as well as various ancillary tests, including flow cytometry, cytogenetic analysis with fluorescence in situ hybridization (FISH), and molecular genetic tests. Core needle biopsy (CNB) is increasingly performed as a primary diagnostic tool for malignant lymphoma, and it has been reported that CNB with larger core needles (19G or larger) tends to yield a greater tissue volume. CNB can provide a sufficient number of specimens, comparable to those obtained with excisional biopsy, while still allowing for an accurate diagnosis [15].

Given the proximity of major vessels, such as the internal thoracic artery, to the tumor and the need for systemic anticoagulation after the biopsy, a 19G×10.5 cm TEMNO Elite introducer needle (Merit Medical Systems, South Jordan, UT) and a 20G×15 cm TEMNO Elite semi-automatic biopsy needle (Merit Medical Systems) were selected. The TEMNO Elite needle was evaluated by comparison with the earlier TEMNO Evolution (Merit Medical Systems) in CT-guided lung biopsies. Samples obtained with the TEMNO Elite showed less fragmentation, improved yield, better macroscopic appearance, and higher tumor content (>30%), although without statistical significance due to the limited sample size [16]. Based on these results, the TEMNO Elite was selected to strike a balance between tissue adequacy and needle size.

One limitation of this strategy is that it may not be applicable to cases where the tumor is unresponsive to chemotherapy. In the present case, the clinical background strongly suggested a diagnosis of lymphoma or germinoma, justifying the decision to proceed with this approach.

## Conclusions

We successfully achieved prompt treatment of a massive anterior mediastinal tumor causing respiratory and circulatory failure using a multidisciplinary approach, including ECMO support and rapid CNB diagnosis. Although our strategy may not be appropriate in tumors unresponsive to chemotherapy, it may be effective in cases involving chemosensitive tumors such as lymphoma. This report highlights the practical steps and clinical strategies that may guide the management of similar cases in emergency or critical care settings.
